# Sodium selective erythrocyte glycocalyx and salt sensitivity in man

**DOI:** 10.1007/s00424-014-1577-0

**Published:** 2014-07-17

**Authors:** Hans Oberleithner

**Affiliations:** Institute of Physiology II, University of Münster, Robert-Koch-Str. 27b, 48149 Münster, Germany

**Keywords:** Glycocalyx, Sodium sensitivity, Salt blood test, Surface charge, Hawthorn, Polyphenol

## Abstract

Negatively charged surfaces of erythrocytes (RBC) reflect properties of the endothelial glycocalyx. Plasma electrolytes counteract these charges and thus control the repulsive forces between RBC and endothelium. Although Na^+^ is supposed to exert a rather high affinity to the RBC surface, a direct comparison between Na^+^ and K^+^ in counteracting the RBC surface has been never made. Therefore, we measured Na^+^/K^+^ selectivity of the RBC surface in 20 healthy volunteers applying the previously published salt blood test (SBT). It turned out that the Na^+^/K^+^ selectivity ratio of the RBC glycocalyx is on average 6.1 ± 0.39 (ranging from 3 to 9 in different individuals). Considering standard plasma Na^+^ and K^+^ concentrations, binding probability of Na^+^/K^+^ at the RBC surface is about 180:1. The SBT reveals that plasma K^+^ counteracts only about 7 % of the negative charges in the RBC glycocalyx. As an in vivo proof of principle, a volunteer’s blood was continuously tested over 6 months while applying a glycocalyx protective polyphenol-rich natural compound (hawthorn extract). It turned out that RBC Na^+^ sensitivity (the inverse of Na^+^ buffer capacity) decreased significantly by about 25 % while Na^+^/K^+^ selectivity of the RBC glycocalyx declined only slightly by about 8 %. Taken together, (i) plasma Na^+^ selectively buffers the negative charges of the RBC glycocalyx, (ii) the contribution of K^+^ in counteracting these negative surface charges is small, and (iii) natural polyphenols applied in vivo increase RBC surface negativity. In conclusion, low plasma Na^+^ is supposed to favor frictionless RBC-slipping through blood vessels.

## Introduction

In humans, one liter of blood contains about one billion erythrocytes (RBC) that usually neither lump together nor attach to blood vessel walls. This impressive phenomenon is explained by the negatively charged (repulsive) surfaces of RBC and vascular endothelium [17, 28]. The surfaces are coated with a gel-like glycocalyx, rich in water and anionic glycosaminoglycans [23, 31]. Surface electronegativity creates so-called zeta potentials that are counteracted by plasma cations [11, 12]. In this scenario, Na^+^ plays a major role due to its high concentration in the extracellular fluid (e.g., blood), its high affinity to the negatively charged surfaces of RBC and endothelium, and its damaging influence on the glycocalyx at excessive plasma concentrations [3, 18, 20].

Recently, we described the so-called salt blood test (SBT) that characterizes in quantitative terms the Na^+^ binding properties of RBC surfaces [21]. Since RBC surfaces “mirror” those of the vascular endothelium, the RBC surfaces reflect, at least to some extent, properties of endothelial surfaces [17]. Since not only plasma Na^+^ but also plasma K^+^ is known to significantly alter vascular endothelial function [19], the question was raised on the role of K^+^ in the generation of the zeta potential and glycocalyx conformation. Therefore, the SBT, aimed to address Na^+^ sensitivity, was expanded including K^+^ as another inorganic cation. In particular, we focused on the selectivity ratio, Na^+^/K^+^, in terms of counteracting the negative RBC surface charges. The latter were evaluated by measuring RBC sedimentation velocity in different electrolyte solutions. Furthermore, as a proof of principle, we tested whether Na^+^/K^+^ selectivity changes when the Na^+^ buffering capacity at the RBC glycocalyx is increased by polyphenol-rich hawthorn extracts. These natural compounds are known to act on the vascular system [2, 4], possibly directly on the endothelial glycocalyx [22]. Here, we describe that, in comparison to K^+^, RBC glycocalyx is highly selective for Na^+^ and that any improvement of the glycocalyx is likely to increase Na^+^ buffering capacity independent of ambient K^+^.

## Methods

### Expanded salt blood test

**Fig. 1:**
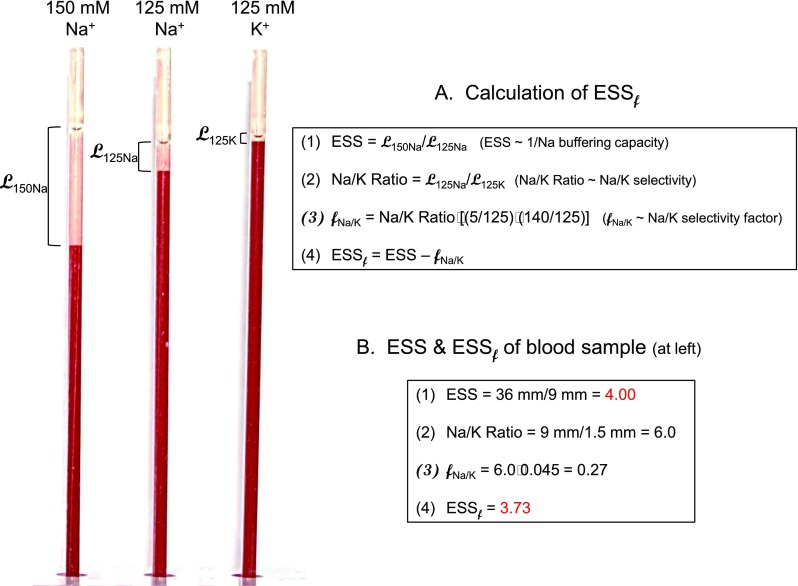
Salt blood test (SBT) and calculations **a** leading to ESS (erythrocyte sodium sensitivity), the Na^+^/K^+^ selectivity factor (*f*_Na+/K+_), and ESS_*f*_ (ESS–*f*_Na+/K+_) and **b** leading to representative ESS and ESS_*f*_ values taken from the three glass capillaries (shown on the *left*)

The SBT has been described in detail previously [21]. The SBT was expanded in a way allowing—in addition to the evaluation of erythrocyte sodium sensitivity (ESS)—measurements of RBC Na^+^/K^+^ selectivity. In short, 4 ml of blood is drawn from volunteers by venous puncture using heparinized monovettes (Sarstedt Company, Sarstedt, Germany). Blood is centrifuged and washed twice in buffered electrolyte solution (in mmol/l: 10 HEPES = 4-(2-hydroxyethyl)piperazine-1-ethane sulfonic acid, 140 NaCl, 5 KCl, 1 CaCl_2_, 1 MgCl_2_; 1 % bovine serum albumin, pH 7.4). For a single measurement, washed RBC (50 μl) are suspended in three Eppendorf vials containing 75 μl of either 150 mmol/l NaCl, 125 mmol/l NaCl, or 125 mmol/l KCl (fixed hematocrit 0.4). All three solutions contain 3 % dextran (Sigma 44886, MW 70,000 D). In addition, sucrose is added for maintaining constant osmolality as appropriate. Hematocrit capillary tubes (Safecap P75-2,000 M; length 75 mm; Scholz Company, Neubiberg, Germany) are filled by capillary forces with the respective three RBC suspensions. Hematocrit capillary tubes, closed at the lower end, are put on stands in an upright position (Fig. 1). RBC sedimentation (i.e., the length of the supernatant) is measured after 60 min. Erythrocyte sodium sensitivity (ESS) of the individual blood samples is calculated as the ratio of the respective supernatant lengths in 150 and 125 mM Na^+^ (ESS = *L*_150Na_/*L*_125Na_). Na^+^/K^+^ selectivity is calculated as the ratio *L*_125Na_/*L*_125K_. Calculations of the selectivity factor *f*_Na+/K+_ and ESS_*f*_ (ESS modified by *f*_Na+/K+_) are documented in Fig. 1 and illustrated by a representative example.

### Hawthorn experiments in vivo

After a control period of several weeks, a healthy volunteer ingested 450 mg of hawthorn extract (Crataegutt novo® 450 mg, Schwabe GmbH, Karlsruhe, Germany) daily. ESS values and Na^+^/K^+^ selectivity were evaluated over a period of about 6 months. This experimental series was preceded by a pilot series of similar length but without measuring Na^+^/K^+^ selectivity. Since a clear effect of hawthorn extract on ESS was discovered in this first pilot series (not shown), a full-blown series was started after a break of 3 months (no medication) on which it is reported here. Data in Fig. 4a–c are mean values of at least three measurements of the same blood sample taken at the respective days after start of treatment.

## Results

**Fig. 2:**
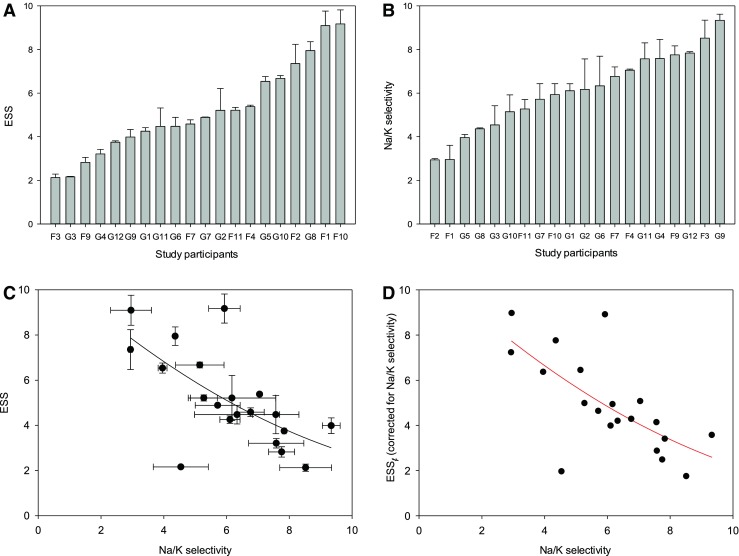
**a** Erythrocyte sodium sensitivity (ESS) and **b** Na^+^/K^+^ selectivity of the erythrocyte glycocalyx measured in 20 healthy volunteers (labeled by *code numbers*). **c** ESS measured as a function of glycocalyx Na^+^/K^+^ selectivity. The correlation is statistically significant (Spearman correlation coefficient = 0.70; *p* < 0.001). **d** Erythrocyte sodium sensitivity corrected for glycocalyx Na^+^/K^+^ selectivity (ESS_*f*_) measured as a function of Na^+^/K^+^ selectivity. The correlation is statistically significant (Spearman correlation coefficient = 0.71; *p* < 0.001). In **a**, **b**, and **c**, mean values of measurements of the same blood sample are given ± SEM (*n* = ≥3)

**Fig. 3:**
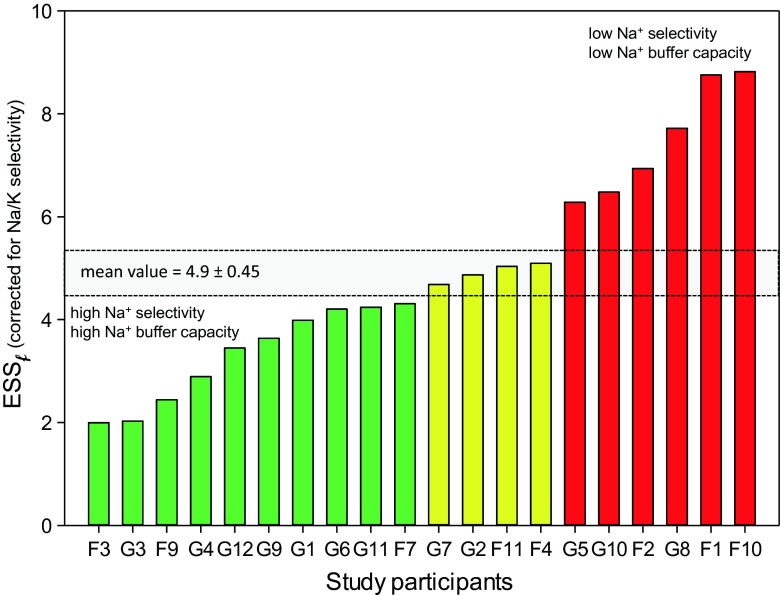
Erythrocyte sodium sensitivity corrected for Na^+^/K^+^ selectivity (ESS_*f*_) measured in 20 healthy volunteers (labeled by *code numbers*). *Red columns* high sodium sensitivity, *green columns* low sodium selectivity, and *yellow columns* moderate sodium sensitivity

Figure 1 shows a representative example of a single measurement. From the lengths of the respective supernatants, ESS values and Na^+^/K^+^ selectivity ratios are calculated. In order to determine a plasma Na^+^/K^+^ selectivity factor *f* which considers standard plasma Na^+^ (140 mmol/l) and K^+^ (5 mmol/l), the Na/K ratio is modified by relating the experimental Na^+^ and K^+^ concentrations (150/125 mmol/l Na^+^ and 125 mmol/l K^+^) to plasma values. As apparent from Fig. 1, the ESS value decreases by only about 7 % when Na^+^/K^+^ selectivity is taken into account. Figure 2a shows ESS values of 20 healthy volunteers (average age = 23 years). Each blood sample was analyzed at least three times. ESS values above 6 indicate a high sodium sensitivity; values below 6 indicate a moderate to low sodium sensitivity. Figure 2b displays the Na^+^/K^+^ selectivity of the same individuals. On average, the RBC glycocalyx exerts a six times higher affinity to Na^+^ as compared to K^+^. As indicated in Fig. 2c, there is a significant correlation between ESS and Na^+^/K^+^ selectivity. In other words, the RBC glycocalyx of individuals with high Na^+^ sensitivity exerts a low Na^+^/K^+^ selectivity and vice versa. By using these Na^+^/K^+^ selectivities, modified ESS_*f*_ values can be calculated. As shown in Fig. 2d, the ESS_*f*_ values (where K^+^ is taken into account) differ only slightly from the “raw” ESS values (where K^+^ is not taken into account; Fig. 2c). Figure 3 displays the modified ESS_*f *_values which can be compared with the “raw” ESS values shown in Fig. 2a. These values are similar as expected. Taken together, the presence of K^+^ has only little influence on RBC Na^+^ sensitivity indicating that Na^+^ is the dominating counteracting cation of the negatively charged RBC glycocalyx.

**Fig. 4:**
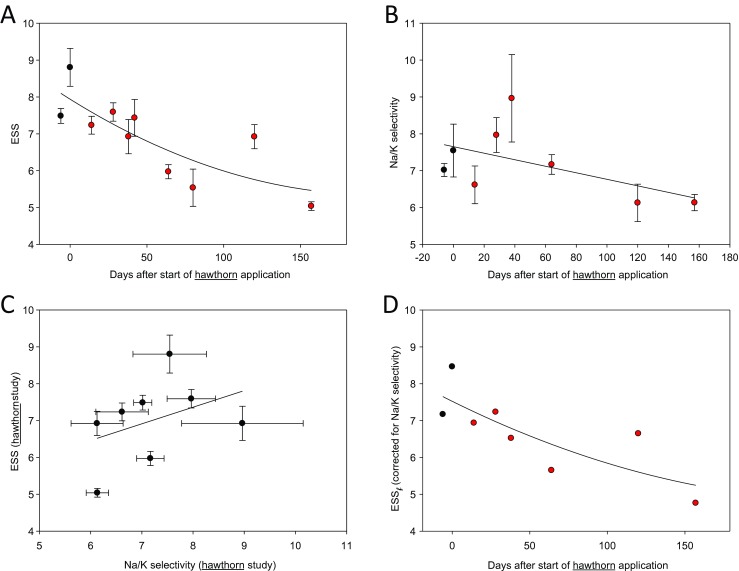
Data of the hawthorn study. **a** Erythrocyte sodium sensitivity (ESS) and measured over a total period of about 6 months applying hawthorn extract (oral dose 450 mg/day) in a single individual. The decrease of ESS over time (∼25 %) is statistically significant (Spearman correlation coefficient = 0.85; *p* < 0.002). **b** Erythrocyte Na^+^/K^+^ selectivity measured over the same time period. The decrease of Na^+^/K^+^ selectivity over time (∼13 %) is statistically not significant (Spearman correlation coefficient = 0.38; *p* = 0.35). **c** ESS measured as a function of glycocalyx Na^+^/K^+^ selectivity. The correlation is weak but significant (Spearman correlation coefficient = 0.15; *p* = 0.049). **d** Erythrocyte sodium sensitivity corrected for Na^+^/K^+^ selectivity (ESS_*f*_) measured over time. The decrease (about 27 %) of ESS_*f*_ over time is statistically significant (Spearman correlation coefficient = 0.81; *p* < 0.02). In **a**–**c**, mean values of measurements of the same blood sample are given ± SEM (*n* = ≥3). *Filled black symbols* in **a**, **b**, and **d** indicate the control period (2 weeks prior to hawthorn application)

Previous studies indicate that hawthorn extract improves glycocalyx function in vascular endothelium [22] and, in parallel, decreases erythrocyte sodium sensitivity [21]. Since the experiments of the former studies were performed in vitro, it was tempting to test whether hawthorn extract influences ESS in a long-term experiment in vivo and whether there is any change in the Na^+^/K^+^ selectivity over time. In a series of experiments, blood of a single healthy individual was continuously analyzed over a period of about 6 months. Figure 4a shows the ESS values over time. There is a gradual decrease in sodium sensitivity (ESS values) reaching about 25 % after 6 months. At the same time, there is also a minor (statistically not significant; *p* = 0.35) decrease in Na^+^/K^+^ selectivity over time (Fig. 4b). As indicated in Fig. 4c, there is a weak correlation between ESS and Na^+^/K^+^ selectivity (*p* = 0.049). When the respective Na^+^/K^+^ selectivity values are taken into account, a modified ESS, namely ESS_*f*_, can be calculated (Fig. 4d). Comparing the results of Fig. 4a (ESS values versus time) with those of Fig. 4d (ESS_*f*_ values versus time), it is apparent that the slight decrease of the Na^+^/K^+^ selectivity in response to hawthorn treatment does not significantly influence erythrocyte sodium sensitivity. In other words, the polyphenol-rich hawthorn extract improves the RBC glycocalyx Na^+^ buffering power independent of ambient K^+^.

## Discussion

It was observed previously that the glycocalyx of erythrocytes and vascular endothelium interact with each other [17]. This interaction strongly depends on the negative charges of the glycocalyx [8, 25]. Any loss of negative charge of either endothelium or erythrocytes gradually damages the respective membrane surfaces [18]. These observations led to the development of the SBT aiming to evaluate the erythrocyte surfaces and thus getting insight into the quality of the vascular glycocalyx. The focus is on the interaction of Na^+^ with the erythrocyte surfaces since this ion species is dominating the extracellular fluid including blood, and there is strong evidence in the literature that the Na^+^ affinity of the endothelial glycocalyx is supposed to be high [3, 26]. Furthermore, excessive extracellular Na^+^ concentrations alter endothelial function in terms of reduced nitric oxide release [7] and lead to damages of the endothelial glycocalyx [20]. Since K^+^ is supposed to be a “vascular protective” ion as derived from in vitro experiments [19] and also from clinical studies [1, 9], it was of considerable interest to characterize the affinity of K^+^ to the glycocalyx.

The results of the present study show that the affinity of K^+^ is about six times less than that of Na^+^, and given the typical plasma concentrations of Na^+^ and K^+^, the binding probability of K^+^, or better the probability of K^+^ to counteract the negative charges of the glycocalyx, is only about 0.5 % compared to that of Na^+^. This indicates that K^+^ does not counteract the negative surface charges to a significant extent indicating that this ion species cannot be made responsible for any significant decreases in RBC zeta potentials and thus will not contribute to enhanced interactions between RBC and vascular endothelium in vivo.

### Potential mechanism underlying high glycocalyx Na^+^/K^+^ selectivity

The question arises why Na^+^ interacts so strongly with the RBC glycocalyx while K^+^ interaction is comparably weak. A similar phenomenon, namely a high Na^+^/K^+^ selectivity, has been described for protein surfaces [30]. There, the preference of Na^+^ over K^+^ was explained mainly by cation-specific interactions with the side-chain carboxylate groups of the respective proteins including actin, RNAse, and some others. Obviously, ion specificity originates from local interactions with charged and polar groups at the protein surface [29]. Possibly, a marked difference between Na^+^ and K^+^ affinities to the glycosaminoglycans of the glycocalyx lies in the different physicochemistry of the two ion species [5, 6, 13, 32]. Na^+^ is a small ion (radius = 102 pm) with a surface area of 131 pm^2^. Its relative positive surface charge is 1.06. Due to its rather high charge density, Na^+^ binds water molecules tightly. Na^+^ is a marginally kosmotropic ion, a so-called “water structure maker.” In contrast, K^+^ is larger compared to sodium (radius = 138 pm) with a surface area of 239 pm^2^. Its relative positive charge is 0.59. Due to its rather low charge density, K^+^ binds water molecules weakly, at least compared to Na^+^. K^+^ is a weakly chaotropic ion, a so-called “water structure breaker” [10]. K^+^ disturbs hydrogen bounds and the water structure in its vicinity. Relating these ion characteristics to the functional properties of the RBC glycocalyx, some assumptions can be made. More than 90 % of the glycocalyx consists of water in which the negatively charged proteoglycans are embedded [24]. Due to the large charge density of Na^+^, this ion species is preferentially attracted by the glycocalyx surface. In addition, Na^+^ as a “water maker” attracts the polar water molecules to a larger extent as compared to K^+^ [10]. Taken together, Na^+^ in comparison to K^+^ dominates the function of the glycocalyx. In case of excessive plasma Na^+^, however, the zeta potential decreases under a (still unknown) threshold value leading to enhanced RBC aggregation and endothelial damage [11, 12].

### In vivo application of Hawthorn extract—a case report

In order to test whether the RBC Na^+^/K^+^ selectivity is altered when changing the glycocalyx properties, an in vivo experiment was performed. Previous studies indicate that polyphenol-rich hawthorn extract improves the nanomechanical glycocalyx of endothelial cells in culture [22]. Furthermore, it was shown in vitro that the interaction between RBC and endothelium is attenuated by this natural compound [21]. Here, an experimental series (case report) in vivo shows that hawthorn application over a period of 6 months lowers the ESS value indicating a decrease in sodium sensitivity or, in other words, an increase of the glycocalyx Na^+^ buffering power. Obviously, hawthorn extract (i.e., most likely the polyphenols of the extract) leads either to (quantitatively) more negative surface charges and/or to a (qualitative) change in glycocalyx conformation that leads to an improved exposure of the negative charges on the cell surface. Indeed, a better exposure of the anionic viscoelastic biopolymers (glycocalyx) due to shear stress is supposed to preferentially bind Na^+^ on the filamentous glycosylaminoglycans as postulated previously for vascular endothelium [27].

Na^+^/K^+^ selectivity slightly decreases along the course of the hawthorn experiment. This small decrease has no strong impact on the ESS values. Taken together, although Na^+^/K^+^ selectivity can vary widely between individuals, between 3 and 9 (this study), it has no considerable influence on glycocalyx Na^+^ buffering power. Improvement of the glycocalyx by applying hawthorn extracts increases the Na^+^ buffering capacity (i.e., decreases the ESS value). Nevertheless, it cannot be excluded that K^+^ interferes significantly with glycocalyx function in certain physiological or pathological conditions. Physical exercise can lead to high local K^+^ concentrations (≥10 mmol/l) in the interstitium of skeletal muscle [15, 16]. Similar may happen during enhanced activities in limited areas of the central nervous system [14]. Then, K^+^ could increasingly participate in the counteraction of the negative surface charges. The question whether such conditions have any impact on RBC aggregation and RBC-endothelial interaction is still open.

### Clinical perspectives and speculations

Negatively charged RBC surfaces attract sodium ions (and water) forming a “safety cushion” between individual RBC but also between RBC and endothelial surface. The ESS value evaluates this cushion. Large ESS values (>5) indicate rather thin cushions; low ESS values (<5) indicate rather thick cushions. Natural polyphenols may thicken (swell) the glycocalyx and thus improve its “cushion” function. A low ESS value indicates “good” Na^+^ buffering power in the blood vessel system. Such an individual is less sensitive to ingested Na^+^ and possibly protected more efficiently against any vascular damage. This assumption, however, needs to be tested in clinical trials.
